# Impact of Obesity on Immunity to the Influenza Virus: Gut Microbiota, Mechanisms, and Novel Therapeutic Strategies

**DOI:** 10.3390/diseases13080267

**Published:** 2025-08-19

**Authors:** Xiaoyue Ji, Jing Sun

**Affiliations:** 1 Institute of Comparative Medicine, College of Veterinary Medicine, Yangzhou University, Yangzhou 225009, China; dx120200169@stu.yzu.edu.cn; 2 Jiangsu Co-Innovation Center for Prevention and Control of Important Animal Infectious Diseases and Zoonosis, Yangzhou University, Yangzhou 225009, China

**Keywords:** influenza virus, obesity, gut microbiota, immunity

## Abstract

Obesity affects millions of individuals globally, and a deeper understanding of its associated physiological disturbances is essential for addressing key public health concerns. It has been demonstrated that the influenza virus possesses substantial global epidemic potential, with higher incidence rates observed in obese individuals and prolonged recovery times. Obese individuals exhibit impaired immune organ function, decreased immune cell activity, disrupted metabolism characterized by mitochondrial dysfunction, and an imbalance in gut microbiota associated with intestinal mucosal barrier damage. The gut microbiota and their metabolic composition in obese patients differ from those in non-obese individuals, potentially promoting viral replication and exacerbating disease severity. These factors collectively contribute to more severe tissue damage and heightened immune responses in obese patients during influenza infection. Therefore, understanding the impact of obesity on influenza virus infection dynamics enables the development of strategies promoting healthy lifestyles to manage body weight and enhance immunity against viral infections. Additionally, given that this special population may not respond optimally to antimicrobial drugs and vaccination, it is necessary to consider how treatment strategies for this group are managed. This review illustrates findings concerning the impact of obesity on the immune response to influenza virus infection, including potential underlying mechanisms.

## 1. Introduction

The influenza virus is a highly contagious pathogen that imposes a considerable socioeconomic burden of morbidity and mortality globally. Epidemiological reports show that periodically occurring influenza pandemics can result in higher mortality rates compared to seasonal outbreaks [1,2]. Since the outbreak of the H1N1 Spanish influenza in 1918 [3], numerous influenza pandemics have occurred, such as the H2N2 Asian influenza in 1957 [4], the H3N2 Hong Kong influenza in 1968 [5], and the H1N1 influenza pandemic in 2009 [6]. Since then, influenza virus infection has been responsible for numerous global pandemics, resulting in tens of millions of deaths and extensive lung injury, suggesting that the virus is significantly associated with severe respiratory pathology.

Obesity is the product of the combined influence of biological and environmental factors, resulting in an increase in excess adipose tissue in the body, which leads to increased morbidity and mortality of certain diseases [7]. Despite the implementation of nutritional interventions and physical education programs, the global prevalence of obesity continues to increase, with the World Health Organization estimating that 20% adults worldwide will be overweight by 2035 [8]. The most recent data from the “China Nutrition and Chronic Disease Status Report” indicates that the prevalence of overweight and obesity among Chinese residents aged 18 and above has surpassed 50%, with the obesity rate among children and adolescents aged 6 to 17 approaching 20%, and the obesity rate among children under 6 years old reaching 10% [9]. The data reflect a significant increase in the risk of chronic diseases, such as cardiovascular disease, diabetes, and cancer. In particular, the risk of developing diabetes increases by 27% for every 5 kg gained [10]. Although nutritional status has long been recognized as a critical determinant of the body’s response to infections, weight increase in the progression and transmission of infectious diseases has recently gained increasing attention. It has been documented that H1N1 can replicate in human primary adipocytes, indicating that obesity may serve as an independent risk factor for increased severity of influenza infection [11]. Therefore, considering the escalating obesity epidemic and its association with influenza complications, it is essential to investigate the key metabolic, gut microbiota, and immune dysregulations during influenza virus infection, as well as their impact on promoting intra-host viral diversity.

## 2. The Epidemiological Association Between Obesity and Influenza Infection

**Figure 1:**
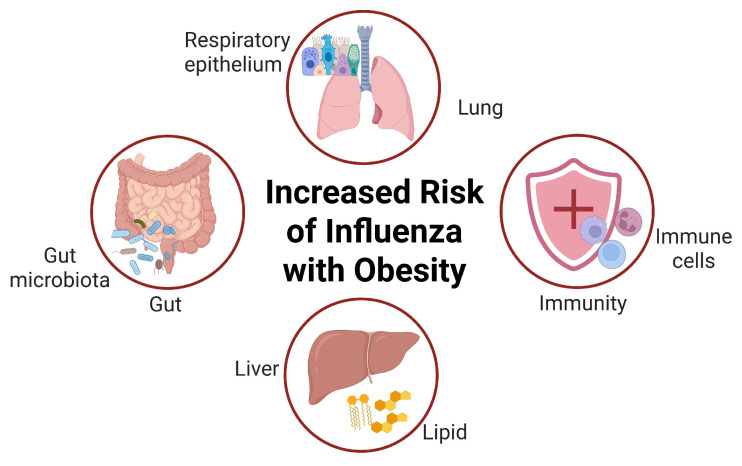
Obesity increases the body’s susceptibility to influenza virus infection. Obesity can influence the host’s resistance to the influenza virus in multiple aspects, including the lung, liver, intestines, and immunity. Obesity is associated with impaired lung function. It elevates the activity of liver lipid synthesis enzymes and blood free fatty acid levels, which, in turn, facilitates virus replication. Additionally, obesity modifies gut microbiota and metabolite levels, damages intestinal tight junction proteins, hinders T cell maturation, and inhibits B cell function. Collectively, these effects lead to the dysregulation of immune function, thereby promoting influenza virus infection. Created in BioRender. Xiaoyue Ji. (2025), https://app.biorender.com/illustrations/6817c8d9ea63ffa10afbeab0, accessed on 25 June 2025.

Obesity has been associated with a variety of respiratory disorders, such as chronic obstructive pulmonary disease, asthma, pulmonary embolism, and aspiration pneumonia. An in-depth data analysis has shown that obesity was an independent risk factor linked to morbidity and mortality during the influenza A (H1N1) pandemic [12]. Concretely, obesity can influence the host’s resistance to the influenza virus in multiple aspects, including the lung, liver, intestines, and immunity (Figure 1). Obesity can reduced lung volume, abnormalities in respiratory muscle function, and impaired gas exchange [13]. Impaired lung function significantly promotes influenza virus infection and subsequent lung injury, which are further aggravated by sleep apnea and chronic inflammation [14]. In obese patients, the activity of lipid synthesis enzymes in the liver is markedly elevated, and the breakdown of adipose tissue also increases. This results in a high concentration of free fatty acids in the blood, which provides raw materials for virus replication [15]. The virus commandeers lipid metabolism enzymes (such as DGAT1) to synthesize viral envelope lipids, increasing the amount of virus released by 3–5 times [16]. Obesity-related high-fat diets and inflammatory factors (such as TNF-α) can damage the tight junction proteins (such as ZO-1 and Claudin-1) between intestinal epithelial cells, leading to increased intestinal permeability [17]. The gut microbiota of obese patients mainly consists of Firmicutes and Proteobacteria, while beneficial bacteria, such as Bacteroidetes and butyrate-producing bacteria (Roseburia and Oscillibacter), are significantly reduced, causing a decrease in the levels of butyrate, acetate, and propionate in the intestine. These metabolites have anti-inflammatory and immune-regulating effects [18]. Obesity impedes T cell maturation, weakens the adaptive immune response, and also inhibits B cell function, resulting in the dysregulation of the antiviral innate immune function [19]. All these factors render obese individuals more vulnerable to influenza virus infection, and the symptoms after infection are more severe than those of the general population.

Higher BMI has been identified as an additional risk factor for hospitalization. Clinical evidence demonstrates that the degree of adiposity correlates with disease severity following infection [20]. Influenza viral RNA has been detected in the aerosols with a positive correlation observed between aerosol viral load and BMI [21]. In addition to the infections mentioned before, increased BMI has been positively correlated with adiposity in the airway wall, wall thickness, and inflammation, which can lead to asthma-related deaths [22].

During influenza season, the hospitalization incidences and hospital stays among obese individuals were found to increase, suggesting that obesity hinders the recovery process [23]. Furthermore, obesity delays the clearance of the influenza virus load and extends the shedding duration, thereby leading to long-term transmission [24]. A severe case confirmed that obese patients infected with the H1N1 virus have a two-fold greater likelihood of being admitted to the intensive care unit (ICU) [25]. A multicenter study involving 144 ICUs in Spain indicated that obesity was linked to higher ICU resource consumption and longer hospitalizations among individuals infected with H1N1 [26]. Among patients admitted to the ICU because of H1N1 infection, obese and morbidly obese patients are more prone to developing pneumonitis than non-obese patients [27]. Even younger patients over the age of 20 years with underlying co-morbidities, such as obesity and diabetes, had a higher hospitalization rate and mortality [12,28,29]. In addition, obesity rates of up to 6.8% have been found among the pediatric patients hospitalized due to influenza, including young children aged under 5 years [30]. Fever is the main symptom in up to 90% of elderly patients and 97–99% of children, lasting approximately 5 days [31]. Individuals who are obese also frequently experience fever during influenza, and the duration of the fever is notably longer. Thus, data from the influenza pandemic indicate that obesity poses a risk regardless of age. However, the exact mechanism by which obesity affects the influenza virus is not yet clear. We summarized some potential new protein targets associated with obesity that exacerbate influenza infection in respiratory disease, including α/β-hydrolase domain 6, Histone deacetylase 6, G-protein-coupled receptor 4, and so on, which can affect obesity and influenza (Table 1).

## 3. The Relationship Between Obesity and Gut Microbiota

Obesity not only leads to an increased hospitalization rate and prolonged hospital stays among influenza patients but also causes intestinal flora imbalance in these individuals. The association between obesity and gut microbiota has been investigated in animal models and human patients. Since the early stages of human microbiome research, a remarkable difference in the composition of the gut microbiome has been detected in obese individuals [42]. Menni et al. pointed out that patients with a more diverse gut microbiota exhibited a lower likelihood of experiencing long-term weight gain, suggesting that a stable gut microbiota may potentially play a role in preventing excessive weight fluctuations, including both gain and loss [43]. Of note, a decrease in *Akkermansia muciniphila* has most consistently been observed to be associated with obesity [44]. Other studies concentrated on the disparities detected in the Bacillota/Bacteroidota ratio among obese and lean individuals [45]. However, the use of the Bacillota/Bacteroidota ratio as a marker for obesity is somewhat controversial. As reviewed, other studies reported discrepancies where obese patients presented a decreased Bacillota/Bacteroidota ratio [46]. Another element contributing to these inconsistencies is linked to the metabolic endotoxemia hypothesis. This hypothesis suggests that elevated adiposity and the development of low-grade inflammation might be associated with the spread of LPS. Obesity is intricately associated with low-grade chronic inflammation. A high-fat diet has the potential to modify the composition of the gut microbiota. This modification results in an elevation of endotoxins in the intestine, subsequently augmenting intestinal permeability. The gut microbiota activates immune signaling pathways by modulating intestinal permeability. This activation elicits chronic, low-grade inflammatory responses, which, in turn, heighten the risk of obesity-related disorders [47]. However, this theory conflicts with the decreased levels of Bacteroidota found in obese patients. The reason is that this phylum mainly consists of Gram-negative bacteria, which are rich in LPS [46]. These discrepancies might also be associated with the experimental procedures employed in various studies, such as the number of participants, the methods utilized, and even the heterogeneity among participants.

Furthermore, scientific evidence suggests that gut microorganisms may extract additional energy from the diet, contributing to the link between gut microbiota and obesity. The gut microbiota breaks down certain dietary compounds that would not be digested otherwise, like some carbohydrates and fibers. Subsequently, the intestinal microbiota transforms these compounds into short-chain fatty acids (SCFAs). SCFAs account for 10% of the daily energy intake of humans and are crucial for colon and liver cells. It has been discovered that when both lean and obese adults consume a diet with an excessive calorie amount, lean individuals expel more energy through feces compared to obese individuals [48]. However, a central theme regarding the relationship between obesity and microbiota is that diet significantly influences the composition of the intestinal microbiota, and as a result, affects the host’s health. A Western-style diet, which is high in saturated fats and low in fiber, can trigger changes in gut microbiota. This leads to a decrease in the abundance of beneficial microorganisms (dysbiosis) and an increase in inflammation. Such a pattern is frequently associated with the development of metabolic disorders, like obesity [49]. Therefore, metabolic disorders in obese individuals can disrupt the intestinal microbiota, ultimately resulting in an elevated risk of influenza virus infection.

## 4. The Impact of Immune Dysfunction on Obese Individuals

Recent studies have demonstrated alterations in immune cell function in obese individuals compared to those with a healthy weight [50]. Compared to individuals with a normal weight, obese individuals exhibit abnormal distributions of white blood cell subpopulations, along with reduced phagocytic function and oxidative burst activity in monocytes [51]. Furthermore, obesity also reduces the proliferative capacity of immune cells in response to polyclonal stimulation and significantly upregulates T cell immune checkpoint molecules, such as PD-1 and CTLA-4, thereby inducing a T cell exhaustion phenotype [52]. This state of depletion, combined with accelerated thymic atrophy and reduced T cell diversity, leads to upregulated expression of immune checkpoints and impaired antiviral immunity. Epidemiological studies have confirmed that obesity is an independent risk factor for severe influenza, with obese patients exhibiting higher risk of hospitalization and ICU admission [53]. Another experimental study indicates that alveolar macrophage function is impaired, and CD8+ T cells exhibit enhanced influenza virus pathogenesis, leading to prolonged viral shedding in obese mice [54]. These findings are consistent with the conclusion that obesity generally elevates the risk of infection.Table 2 lists some biomarkers for immune competence in obese individuals.

## 5. Mechanisms of the Interaction Between Gut Microbes and the Respiratory Immune System

Obesity not only affects influenza virus infection through metabolic disorders, but also indirectly increases the risk of virus invasion by reshaping the structure of intestinal flora. The gut microbiota is the “second brain” of the host immune system, and its imbalance may break the gut–lung axis, leading to systemic immunosuppression and local mucosal defense defects.

The gut microbiota can regulate respiratory immune responses in two primary ways. First, it generates systemic factors that influence the airways. Second, it directs the recirculation of immune cells from the gut, thereby modulating immune responses in the lungs. For instance, when type 2 innate lymphoid cells (ILC2s) are activated either by IL-25 or as a result of gut helminth infection, they migrate to the airways, thereby facilitating both tissue repair and immune responses against helminths [65]. Microbial colonization in early life also leads to the seeding of invariant natural killer (NK) T cells in both the gut and lungs. These cells offer long-term protection against inflammatory bowel disease (IBD) and allergy [66]. The migration of immune cells to the lung, which is also known as homing, is intricate and mainly relies on inflammatory signals [67]. Interestingly, at the steady state, the homing of immune cells is affected by the circadian rhythm [68]. Even though the microbiota has been demonstrated to have a definite role in the trafficking of immune cells to the gut, it remains uncertain whether the microbiome controls the trafficking of immune cells to the airways under normal, steady-state conditions [69].

Alterations in the gut microbiota can have profound effects on host immune signaling pathways. The interaction between the gut microbiota and the host immune system is complex and bidirectional, involving the mutual regulation of microbial components and host immune responses. A previous study reported that mice fed a high-fat diet were found to have elevated levels of LPS circulating in their bloodstream; however, when administered antibiotics, this increased LPS level was no longer observed [70]. Disruption of the gut barrier by certain factors, such as obesity or pathogenic bacteria, may lead to LPS translocation, whereby the molecule moves through the intercellular junctions of the intestinal barrier into the circulation [71]. Increased intestinal permeability and barrier dysfunction facilitate macrophage infiltration into tissues and the activation of inflammatory cytokines, such as NF-κB, ultimately initiating an inflammatory response [72]. Furthermore, LPS can activate pro-inflammatory pathways by binding to toll-like receptor 4 (TLR-4) on immune cells [73]. Another study demonstrated that flagellin, a structural protein of bacterial flagella present in pathogenic, symbiotic, and commensal bacteria, induces inflammation in intestinal epithelial cells via a TLR5-dependent signaling pathway [74,75]. However, probiotic therapy may reduce gut dysbiosis and intestinal leakage, thereby lowering inflammatory biomarker levels and modulating excessive immune activation [76]. Collectively, the immune system plays a crucial role in maintaining the symbiotic relationship between the host and the microbiota.

It has been reported that the gut microbiota can secrete a diverse array of metabolites that are not inherently present in the human body. Elaborate experiments involving the administration of isotype-labeled bacteria to germ-free (GF) mice revealed that metabolites derived from microorganisms can be detected in nearly all tissues. These tissues include the bone marrow, brain, and adipose tissue. Regrettably, there was no evidence indicating that lung tissue was examined in that study [77]. To further corroborate this phenomenon, it was recently demonstrated that gut-derived butyrate can reach the skin and affect keratinocyte function within 45 min after administration [78]. Research indicates that the gut–lung–immune axis regulates immune function through coordinated interactions among multiple organs, with the hematopoietic process in the bone marrow being one of its key mediators [79,80,81,82]. In addition, the gut microbiota can communicate with distant organs via its structural components and metabolic derivatives. This mechanism also helps explain how the continuous proliferation and lysis of microbial populations result in the release of bacterial components, which can subsequently induce lung injury [83].

Moreover, the gut microbiota exhibits high metabolic activity and synthesizes a wide range of microbial by-products. These microbe-derived molecules are not naturally present in the human body. Conversely, these by-products are absorbed into the peripheral tissues and may interact with the immune system. Before reaching the respiratory tract, they can be further metabolized by the host. Together, the gut microbiota is likely to exert a broader influence on immune homeostasis, which in turn affects influenza infection.

## 6. The Gut–Lung Axis in Influenza Virus Infection and the Immune System

Respiratory viral infections exert bidirectional regulatory effects on the gut microbiota. In turn, the gut microbiota modulates immune defense mechanisms against viral pathogens in the respiratory tract. It has been reported that Influenza A infection can cause dysbiosis in the gut and allow the overgrowth of pathogenic bacteria [84,85]. Although the influenza virus exhibits relatively low replication activity in the gastrointestinal tract, its infection can still modulate the composition of the intestinal microbiota through indirect mechanisms, such as impaired nutrient absorption [85]. This process occurs upon the activation of immune cells in the lung, after which the activated immune cells migrate back to the gut [86]. In recent years, growing evidence has demonstrated that the cluster of differentiation (CD) molecules on effector T cells not only function as immune markers but also contribute significantly to metabolic homeostasis. These cells migrate to non-lymphoid tissues (NLTs) in response to chemokine receptor expression and local chemokine production at infection sites [87]. It was discovered that the production of IFN-γ and IL-17 in the lungs, which was induced by influenza, enhanced the expression of CCR9 on CD4^+^ T cells. This increase enabled the CD4^+^ T cells to migrate to the gut. Once in the gut, they facilitated Th17-mediated intestinal inflammation and tissue damage, ultimately resulting in microbial dysbiosis [86].

In addition to direct microbial translocation, the production of microbial metabolites represents a key pathway associated with the lungs. Among these metabolites, short-chain fatty acids (SCFAs) regulate pulmonary antiviral immunity via multiple mechanisms, particularly by enhancing the metabolic activity and effector functions of systemic CD8+ T cells, thereby improving their capacity to eliminate viruses in the lung [88]. Specifically, butyrate reprograms CD8+ T cell metabolism by enhancing glutamine utilization and fatty acid oxidation, which enhances their immune memory response during secondary infections [89]. Furthermore, environmental factors, such as changes in dietary intake, can indirectly influence SCFA levels. Notably, elevated ambient temperatures have been shown to increase pulmonary autophagy, suppress inflammasome-dependent cytokine secretion, and impair the immune response of virus-specific CD8+ T cells [90]. In addition to the aforementioned findings, other CD markers have demonstrated significant efficacy in combating viral infections. Our review integrates extensive studies that highlight the critical roles played by various T cell-associated CDs during viral infections (Table 3).

A parallel mechanism by which mice supplemented with butyrate or inulin were safeguarded against influenza infection is related to the bone marrow [98]. Additionally, mice fed a high-fiber diet exhibited augmented macrophage–dendritic cell progenitors in the bone marrow [99]. This led to the production of Ly6c patrolling monocytes and an increase in the number of alternatively activated macrophages in the airways. Upon viral exposure, these cells generated less CXCL1. As a consequence, neutrophil infiltration was diminished, and immunopathology was restricted. Nevertheless, this attenuated response of alveolar macrophages might make the immune system more vulnerable to secondary infections. In fact, it has been demonstrated that reduced systemic acetate levels can impair the function of alveolar macrophages, indicating that susceptibility to secondary pneumococcal infection may increase following influenza infection [85].

Type I interferon (IFN) activity represents a critical molecular mechanism by which the intestinal microbiota defends against respiratory viral infections. Under normal conditions, IFNs maintain systemic immune vigilance through positive and negative feedback loops and regulate the production of multiple cytokines [100]. Microbial components activate IFN expression via intracellular RIG-I, cyclic GMP synthase, and TLR signaling pathways. Research indicates that antibiotic-induced depletion of the intestinal microbiota suppresses IFN-associated inflammasome responses, resulting in impaired adaptive immunity and reduced capacity to clear respiratory viruses [101,102,103]. The specific mechanisms include the outer membrane products of Bacteroides species binding to Toll-like receptor 4 (TLR4) on colonic dendritic cells (DCs), inducing the secretion of IFN-β, thereby effectively resisting influenza and other viral infections [104,105]. These microbial components help maintain the basal interferon signaling of the body and enhance the immune defense against viral invasion in the lungs [106]. Firstly, the outer membrane products of Bacteroides can activate colonic DCs through TLR4, promoting the secretion of IFN-β and resisting systemic viral infections, such as influenza [107]. The interferon produced by pDCs can keep conventional DCs in a “pre-activated” state at the metabolic and transcriptional levels, enabling them to activate T cells more rapidly upon antigen stimulation and enhancing the adaptive immune response [108,109]. To date, only a limited number of microbial metabolites or specific microbial species have been identified as direct inducers of tonic IFN production.

A recent study has indicated that in gnotobiotic animals, colonization with Clostridium scindens (expressing the enzyme 7α-dehydroxylase) which is necessary for converting primary bile acids (BAs) into secondary BAs—or direct administration of the secondary bile acid deoxycholic acid (DCA)—can protect mice from systemic chikungunya virus infection [109]. While the precise mechanism by which secondary BAs safeguard against systemic viral infection remains mostly unknown, one proposed pathway involves virus-induced activation of NF-κB. This activation promotes TGR5-mediated cellular uptake and intracellular accumulation of secondary Bas. Subsequently, this accumulation induces the phosphorylation of key antiviral signaling components, facilitating viral clearance [110].

Desaminotyrosine (DAT) is produced by Clostridium orbiscindens during the metabolism of dietary flavonoids and amino acids [111]. DAT can induce tonic IFN signaling and enhance macrophage phagocytic activity, thereby protecting against lethal influenza infection. In addition, some viruses, like SARS-CoV-2, are known to exploit the aryl hydrocarbon receptor (AhR) to evade immune activation, enabling viral replication, triggering mucus production, and dysregulating gas exchange [112,113]. On the contrary, a recent study demonstrated that selectively reducing AhR expression in the lung endothelium protected against influenza-induced pathology, highlighting that the tissue-protective function of AhR plays a dual role at different stages of the disease [114]. These studies prove that microbial metabolites could regulate host immune to influence viral infections, and more metabolites are listed in Table 4.

**Figure 2:**
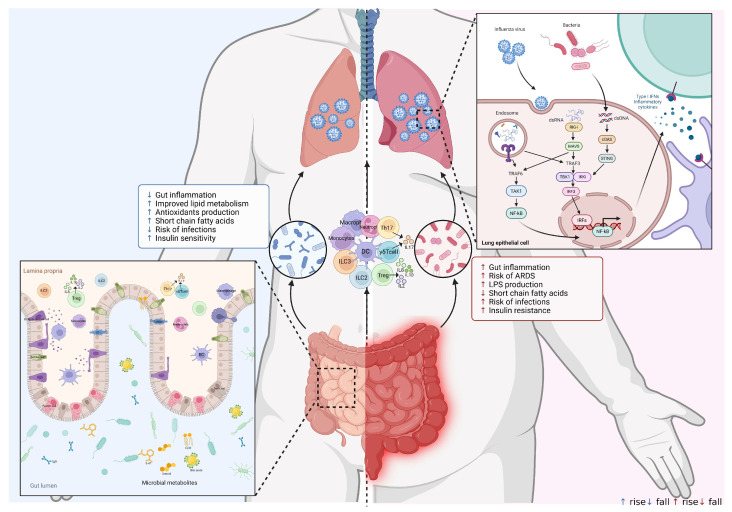
Mechanisms of the gut–lung axis in influenza virus infection. The dsDNA from gut microbiota activates the cGAS-STING signaling pathway, while dsRNA from influenza virus activates the RIG-I-MAVS signaling pathway in respiratory epithelial cells. The dsDNA and dsRNA can phosphorylate and activate TBK1 and its downstream IRF3 transcription factor. The activation of IRF3 dimerizes and enters the nucleus to initiate the transcription of type I interferon genes. The gut microbiota produces metabolites that activate the immune system. In addition, Tregs, Th17 cells, ILC2s, ILC3s, and γδ T cells migrate from the gut to the lungs to impact respiratory immunity. The disorder of intestinal flora in obese individuals is more likely to cause intestinal inflammation and increase the severity of influenza virus infection. Created in BioRender. Xiaoyue Ji. (2025), https://app.biorender.com/illustrations/67ff65b12f92165b5f348839, accessed on 25 June 2025.

Moreover, the gut microbiota can systemically activate the cGAS-STING-IFN pathway, a process that involves the release of membrane vesicles carrying bacterial DNA to immune cells (Figure 2). The lipid encapsulation of microbial DNA facilitates its transport and enables immune modulation at distant anatomical sites, thereby providing protection against respiratory viral infections [124]. It has been demonstrated that a gain-of-function STING mutation model can cause autoimmune vasculopathy and fibrosing lung disease, suggesting that microbiota-driven tonic STING activation may also exacerbate autoimmunity [125]. Furthermore, the gut microbiota regulates IFN production by lung epithelial cells, offering protection against influenza infection. Specifically, the *Clostridium strain C. butyricum* promotes the release of 18-hydroxyeicosapentaenoic acid (18-HEPE), which activates tonic IFN-λ production in lung epithelial cells through the G-protein-coupled receptor GPR120, thereby enhancing resistance to influenza infection [123]. However, some contradictory studies have suggested that IFN-λ production may be detrimental during chronic lung viral infections, indicating that IFN-λ plays a dual role depending on the context [126]. Additionally, short-chain fatty acids (SCFAs), like acetate and butyrate, can trigger an antiviral immune defense against respiratory syncytial virus (RSV) by stimulating airway epithelial cells to produce IFNs [115,116]. In obese patients, the levels of bacterial metabolites, such as lipopolysaccharide (LPS), trimethylamine (TMA), branched-chain amino acids (BCAAs), and indole derivatives, are elevated. Conversely, the concentrations of short-chain fatty acids (SCFAs), including butyrate, acetate, and propionate, along with 18-hydroxyeicosapentaenoic acid (18-HEPE), are decreased [127]. Collectively, these recent studies highlight multiple mechanisms by which gut microbial factors sustain systemic tonic IFN production (Figure 2). Moreover, they demonstrate how these factors regulate the metabolic functions of both innate and adaptive immune cells within the lung, thereby providing protection against respiratory viral infections.

## 7. Novel Therapeutic Strategies Against Influenza Virus Infection

**Figure 3:**
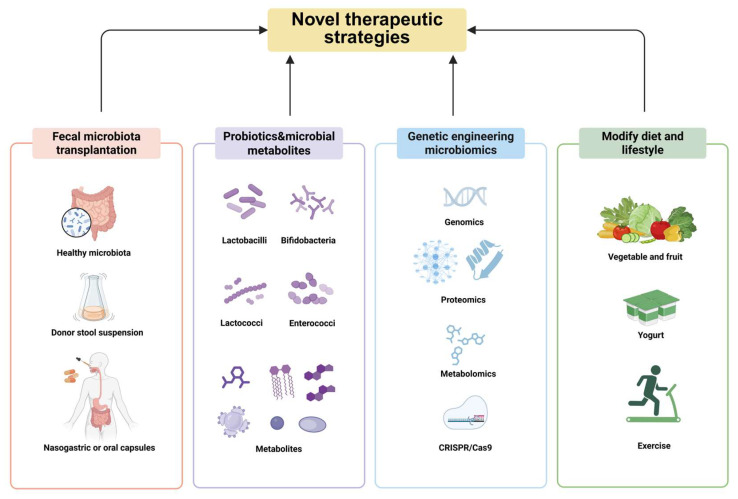
Novel therapeutic strategies targeting the gut microbiota to treat influenza. Fecal microbiota transplantation is beneficial for many diseases, probiotics have adjuvant therapeutic effects for patients infected with viruses, engineered microbes have potential, and dietary intervention can regulate gut microbiota to enhance immunity to resist viral infections. Created in BioRender. Xiaoyue Ji. (2025), https://app.biorender.com/illustrations/681bba8e7395c116bdbb625c, accessed on 25 June 2025.

Influenza A virus results in 3 to 5 million severe cases and 250,000 to 500,000 deaths annually. While vaccination remains the primary preventive strategy, its effectiveness is limited by Influenza A’s high susceptibility to antigenic drift and delays in vaccine production [128]. Moreover, a great deal of information has been reported regarding the resistance to antiviral drugs, such as Oseltamivir and Baloxavir [129]. These limitations underscore the critical need for alternative therapeutic strategies targeting the immune response to influenza virus infection. Intriguingly, recent research has discovered that modulating the gut microbiota can enhance the immune responses against IAV through the gut–lung axis, highlighting that the therapeutic potential of the lung microbiome should be taken into consideration as well [130]. Critical unanswered questions include whether endogenous factors—particularly those derived from the gut microbiome—can regulate its composition and function [131]. The immunotherapy response necessitates further investigation into gut microbiome transplantation, influenced by probiotic abundance and metabolite alterations, to identify specific lung-resident microbes or components with therapeutic potential (Figure 3). Additionally, more studies are required to explore how dietary habits and lifestyle factors affect the gut microbiome.

### 7.1. Fecal Microbiota Transplantation (FMT)

Fecal microbiota transplantation (FMT) is a method that involves transplanting the entire intestinal microbiota of healthy individuals into other patients. As the most direct strategy for regulating the intestinal microbiome at present, FMT can effectively alter the composition of the intestinal microbiota. Multiple studies have confirmed that FMT has beneficial effects on many diseases, such as intestinal graft-versus-host disease, irritable bowel syndrome, inflammatory bowel disease, and multidrug-resistant bacterial infections [132,133,134]. In addition, FMT for the treatment of recurrent *Clostridioides* difficile infection has been included in the clinical guidelines in the United States [135]. Liu et al. conducted oral FMT treatment on 11 discharged patients with COVID-19 to analyze the potential impact of FMT on the intestinal microbiota and immune system after SARS-CoV-2 infection. The results showed that all five patients with gastrointestinal symptoms improved after treatment. FMT corrected intestinal microbiota dysbiosis by increasing the relative abundance of Actinobacteria at the phylum level, reducing the relative abundance of Proteobacteria, and increasing the relative abundance of *Bifidobacterium* and *Faecalibacterium* at the genus level [136]. A case report described two patients with risk factors for severe COVID-19 (Patient 1: an 80-year-old male with pneumonia and sepsis; Patient 2: a 19-year-old male with ulcerative colitis receiving immunosuppressive therapy) who underwent FMT for *Clostridioides* difficile infection and were later diagnosed with COVID-19. Notably, both patients had mild clinical symptoms and did not develop severe COVID-19 but recovered rapidly, suggesting FMT can microbiota homeostasis and promote immune recovery [137]. Together, these studies provide preliminary clues for the application of FMT in the treatment of viral pneumonia, but more research is needed for clinical application.

### 7.2. Targeted Therapy of Probiotics and Microbial Metabolites

Recent studies suggest that probiotics can confer health benefits to the host by enhancing intestinal barrier function, modulating the interaction between the host and microbiota, producing antimicrobial substances and organic acids, resisting pathogens, regulating immune responses, and influencing metabolic processes [138]. Therefore, probiotics can serve as an adjuvant therapy for various diseases, and there are well-established guidelines for their application in pediatric populations [139]. As previously discussed, the gut microbiota in individuals with COVID-19 undergoes significant dysbiosis, much of which is detrimental to host health. Supplementing probiotics to restore intestinal microecological balance shows promise as an adjunctive therapy for COVID-19, potentially enhancing immune function and promoting recovery from infection [140]. A bibliometric analysis of 84 studies on microecological interventions for COVID-19 revealed that probiotics or prebiotics could reinforce the mucosal barrier, regulate the host’s immune system, and enhance the interaction between the gut–lung axis, thereby increasing resistance to SARS-CoV-2 infection and reducing disease duration and severity [141]. Clinically, probiotics have been shown to alleviate symptoms associated with COVID-19, such as diarrhea, fatigue, and fever, while also reducing the need for intensive care [142,143]. Furthermore, microbiota influence disease progression via bioactive metabolites including SCFAs, aromatic compounds, amino acids, bile acids, vitamins, and lipids [144,145]. The human microbiota influences SARS-CoV-2 infection both directly and indirectly through its complex metabolome. Notably, numerous studies have reported a reduced abundance of SCFA-producing bacteria in patients with COVID-19 [146,147]. Zhang et al. identified reduced capacity for SCFA and L-isoleucine biosynthesis in the gut microbiome of COVID-19 patients, alongside increased urea synthesis. This suggests SCFAs play multifaceted roles, including anti-inflammatory effects, immune modulation, and metabolic regulation [148]. In addition to SCFAs, three intestinal microbiota-derived metabolites—tryptamine, 2,5-bis(3-indolylmethyl) pyrazine, and N6-(D2-isopentenyl) adenosine—have been identified. These metabolites function as 5-hydroxytryptamine receptor agonists, demonstrate anti-SARS-CoV-2 activity, and show structural and functional parallels with clinically used antiviral drugs, suggesting targeting probiotics and microbial metabolites may aid in treating viral pneumonia [149]. Based on the aforementioned research, butyrate-producing bacteria can be administered to obese individuals. Moreover, TLR agonists or SCFAs can be incorporated as adjuvants for influenza vaccines to boost the antibody response in obese people.

### 7.3. Transgenic Microbial Therapy

In the past several years, the Food and Drug Administration (FDA) has given approval to a significant number of biologic drugs, including monoclonal antibodies, therapeutic proteins, and vaccines. It has been demonstrated that the large interindividual variation in the composition of the lung and gut microbiomes may reflect functional redundancy. Specifically, metagenomic studies have further emphasized that the host exerts selection pressure based on metabolic functional traits rather than specific taxa, suggesting that future therapeutic strategies should focus on targeting the metabolic output of bacteria rather than their taxonomic classification [150]. Most of these products which are utilized in the treatment of various human diseases are genetically modified and produced through biological pathways. However, the high cost of production, particularly during downstream processing, the short half-life of the drugs, and the limitations in drug delivery collectively hinder their therapeutic applications [151,152]. Thus, engineered microbiomics serves as a promising alternative, with the potential to secrete therapeutic proteins, deliver antigens, monitor the gut environment, eradicate pathogens, modulate the immune system, and metabolize harmful substances [153,154]. It has been reported that the development of a genome-scale library for probiotics using CRISPR can facilitate mapping genotype-phenotype relationships [155]. For instance, Crook et al. found that glycoside hydrolases enhanced cell viability in the gut [156]. Recently, the FDA’s approval of RBX2660 for treating *Clostridioides* difficile infection has paved the way for the market of microbiome-based products. These cases suggest that advances in bacterial genetic engineering technology have made it feasible to modify the intestinal microbiome to enhance resistance to influenza infection. In addition, genetically engineered microorganisms can achieve a higher level of specificity when compared to FMT. In the coming years, many startups, well-established biotechnology companies, and research institutions are expected to make substantial investments in the research and development of live biotherapeutic products for treating human diseases, thereby enriching their product pipelines with bacterial therapies.

### 7.4. Modify Diet and Lifestyle

Dietary intervention can regulate gut microbiota and enhance the efficacy of immunotherapy with minimal harmful risks, suggesting that it may represent a more promising approach. Many diets that have been proven to be beneficial for human health typically contain high levels of dietary fiber. Studies have shown that short-term consumption of diets consisting exclusively of plant products can alter microbial community structure and interindividual differences in microbial gene expression [157]. However, randomized controlled trials of specific foods or nutrients have shown limited long-term effects of these interventions on microbiota and immune function [158]. Gouez et al. have confirmed that severe malnutrition was strongly associated with lower survival [159]. Moreover, Gao et al. found that resveratrol (RESV) can upregulate the cytotoxic effect of CD8^+^ T cells and improve the tumor immunosuppressive microenvironment [160]. In daily life, we can also supplement probiotics by drinking yogurt, which helps to regulate the balance of intestinal flora, promote the digestion and absorption of food, and, thus, maintain the normal physiological function of the intestine [161]. More importantly, we should also focus on strengthening the positive impact of exercise on physical health. Continuous and moderate exercise can effectively enhance the body’s resistance to external bacterial invasions, thereby maintaining a good state of health. At present, dietary adjuvant therapy is still in its infancy, and more experiments are needed to explore the impact of dietary habits and lifestyle on the gut microbiome.

## 8. Conclusions and Future Perspectives

In recent years, the relationship between gut microbiota and immunotherapy has become a hot topic in immunology research. Obesity weakens anti-influenza immunity through multiple mechanisms, and the regulation of the microbiome provides a new target for reversing immune suppression. The composition and function of the gut microbiota are significantly altered in influenza patients. As an important pathway connecting the gut and lung, the regulation of gut–lung axis has a profound impact on the immune microenvironment of the lung. The significance of the microbiota in immunotherapy is assuming an increasingly prominent role. A diverse range of microbial therapies are currently being subjected to clinical trials with the aim of enhancing the efficacy of treatment and the prognosis of patients. Nevertheless, research on the microbiota still confronts certain limitations. Despite active progress in this scientific domain, standardized methodologies and statistical approaches have yet to be firmly established. This lack of standardization renders it arduous to conduct meaningful comparisons across different studies. To the present day, numerous published studies have been constrained by relatively small sample sizes. Additionally, another notable limitation is that the current research predominantly relies on single-sample analyses of either healthy volunteers or patients. Evidently, this approach is inadequate, and longitudinal analyses are also indispensable for a comprehensive understanding. Future research is required to better understand how the composition of the gut microbiome affects the response to immunotherapy and drug resistance, as well as to investigate strategies for enhancing immunotherapy efficacy by restoring microbiome balance, such as through probiotics and FMT. In particular, large-sample and multicenter clinical trials are needed to verify the effectiveness and safety of microbiome intervention and provide reliable basis for clinical practice. Currently, although microbial immunotherapy has been demonstrated to have definite efficacy in treating other diseases (such as lung cancer), only one-third of patients can derive benefits from it. Moreover, the following issues exist: (1) The heterogeneity of gut microbiota donors reduces the comparability and reproducibility of research; (2) the potential pathogenic strains in gut microbiota preparations render its long-term safety uncertain; (3) the dosage, administration method, and administration route of gut microbiota preparations cannot be standardized; (4) a single positively correlated microbial community in the composition of gut microbiota is not a consistent predictor of clinical outcomes; (5) the start time and duration of the antibiotic regimen prior to fecal microbiota transplantation (FMT) cannot be standardized. Although research on the clinical effects of the gut microbiota is still in its early stages, an increasing amount of evidence indicates its indispensable immunomodulatory role. Modifying the composition of gut microbiota is a potential strategy to improve the prognosis of influenza virus patients. With advancements in medicine, it is anticipated that the potential of gut microbiota in influenza immunotherapy will be fully harnessed, leading to improved therapeutic outcomes for patients. Taken together, it is necessary to combine basic research and clinical translation to develop comprehensive treatment strategies based on the microbiota–metabolic–immune axis to reduce the burden of influenza in obese people.

## Figures and Tables

**Table 1:** Potential targets associated with obesity in respiratory diseases.

Target Protein/Pathway	Main Distribution	Function	References
ABHD6	Brain, intestine, and immune system	α/β-hydrolase domain 6 (ABHD6) is a lipase affecting energy metabolism.	[32]
HDAC6	Liver and brain	Histone deacetylase 6 (HDAC6) resensitizes leptin signaling during obesity.	[33]
LGR4	Liver	G-protein-coupled receptor 4 (LGR4) impacts long-chain fatty acid-absorption.	[34]
NK2R	Colon and immune system	Neurokinin 2 receptor (NK2R) can increase energy expenditure peripherally.	[35]
NMDA receptor	Brain	The N-methyl-D-aspartate (NMDA) receptor antagonism can treat obesity.	[36]
PICK1, PSD95	Brain	Protein interacting with C kinase 1 and postsynaptic density protein-95 targeting postsynaptic glutamate receptors for obesity treatment.	[37]
PTER	Liver and brain	Orphan enzyme phosphotriesterase-related (PTER) is a N-acetyltaurine hydrolase.	[38]
PXR	Liver	Pregnane X receptor (PXR) can regulate glycolipid metabolism.	[39]
SREBP2, RORγ	Liver and immune system	Sterol regulatory element-binding protein 2 (SREBP2) and the retinoid acid receptor-related orphan receptor gamma (RORγ) regulate cholesterol metabolism.	[40]
RUVBL2	Liver and brain	Knockout of PVH RUVBL2 results in hyperphagic obesity.	[41]

**Table 2:** An overview of obesity biomarkers and their association with chronic diseases.

Type	Biomarker	Association with Chronic Disease	Function	Signaling Pathway	References
Insulin/IGF axis	Insulin/C-peptide	Cardiovascular disease	Inhibit hepatic gluconeogenesis and promote fat synthesis	GPR146-PLC/PKC-PI3K	[55]
	CRP	Cardiovascular disease, vascular and non-vascular mortality, colorectal cancer	Promote endothelial dysfunction	eNOS	[56]
	IGF-1	Cancer and cardiovascular diseases	Inhibit catabolism	PI3K/AKT/mTOR	[57]
Adipokines	Adiponectin	NASH	Promote fatty acid oxidation	AdipoR1/R2-AMPK/PPARα	[58]
	Leptin	NASH	Increase energy consumption (promote brown fat thermogenesis)	LepR-JAK2-STAT3	[59]
	Resistin	T2DM	Participate in insulin resistance	MyD88-NF-κB	[60]
Immune checkpoint	CD47	Cancer	Reduce metabolic rate	isoQC-pGlu-SIRPα	[61]
	PD-L1	Cancer	Inhibit T cell activation	PD-1/SHP1/2-TCR	[62]
	LAG-3	Cancer	Inhibit inflammation	IL-7Rα	[63]
	Tim-3	Cancer, autoimmune disease	Mediating insulin resistance in adipose tissue	Gal-9-Bat3/c	[64]

**Table 3:** Association between cluster of differentiation and viral infections.

Markers	Cell Types and Distribution	Efficacy	References
CD4	T-cell receptor	CD4 T cells play a multiplicity of roles in protective immunity to influenza, including viral antigen specificity.	[86]
CD8	T-cell receptor	CD8 T cells provide broad cross-reactive immunity and alleviate disease severity by recognizing conserved epitopes.	[90]
CD11	T cells, B cells, monocytes, macrophages, neutrophils, basophilic granulocytes, and eosinophilic granulocytes	CD11b^+^ cDC2 subsets present in mice are regulated by IRF4 during IAV infection.	[91,92]
CD27	Lymphoid cells (naive T cells, activated B cells, NK cells)	CD45RA^−^CD27^−^ effector memory-like T cells are increased in IAV- and IBV-infected patients.	[93]
CD38	Lymphocytes, plasma cells, NK cells, and non-hematopoietic tissues	CD38^+^Ki67^+^CD8^+^ effector T cells are increased in IAV infected pediatric and adult subjects.	[94]
CD45	Hematopoietic cells	The CD45-positive macrophages expressing mCherry are increased in IAV-infected patients.	[91,93]
CD64	Monocyte and macrophage	Mice lacking myeloid TBK1 showed less recruitment of CD64^+^SiglecF^−^Ly6C inflammatory macrophages.	[91]
CD69	T cells, B cells, natural killer (NK) cells, neutrophils, and eosinophils	CD69^+^CD103^+^ T_RM_ cells preferentially localized to lung sites of prior IAV infection.	[95]
CD103	T cells, B cells, lymphocytes, and dendritic cells	Vaccine can induced lung tissue-resident memory T cells expressing high levels of CD103.	[95,96]
CD122	NK cells and activated T cells	Once memory to influenza is established, enhanced NF-κB signaling in T cells can increase CD122 levels.	[97]

**Table 4:** The effect of microbial metabolites in host during virus infection.

Microbial Metabolites	Bacteria	Efficacy	References
Acetate	Acetobacter and *Bifidobacterium pseudolongum*	Acetate can trigger antiviral immunity.	[115,116]
Butyrate	*Clostridium butyricum* and *Butyrivibrio*	Butyrate reprograms CD8^+^ T cells by promoting glutamine utilization and fatty acid oxidation.	[90]
LPS	Gram-negative bacteria	LPS can activate the TLR4 pathway to trigger the NF-κB signaling pathway and regulate the inflammatory response.	[46]
BCAAs	*Prevotellacopri* and *Bacteroides vulgatus*	Branched-chain amino acids can induce insulin resistance.	[117]
Indole derivatives (e.g., IAA, IPA, 5-HIAA)	*Escherichia coli*, *Proteus* and *Vibrio cholerae*	Indole derivatives can activate the AhR.	[118]
5-HT	Enterochromaffin cells produce 5-HT influenced by gut microbiota	5-hydroxytryptaminecan regulates glucose homeostasis.	[119]
PGN	All species of bacteria	Peptidoglycan can activate host immunity.	[120]
2-octagenoate	*Blautia* bacterium	2-octagenoate can lead to liver hypertrophy, steatosis, inflammation of liver cells, and fibrosis.	[121]
DAT	*Clostridium orbiscindens*	DAT can trigger tonic IFN signaling and regulate the phagocytic activity of macrophages.	[111]
TMA	Gut microbiota	Trimethylamine is converted to trimethylamine-N-oxide (TMAO) in the liver. TMAO regulates glucose metabolism and causes adipose tissue inflammation.	[122]
Bile acids	*Clostridium scindens*	BAs activate virus-induced NF-κB.	[110]
18-HEPE	Clostridium strain C. butyricum	18-HEPE activates the production of tonic IFN-λ by lung epithelial cells via GPR120, leading to enhanced resistance to influenza infection.	[123]

## Data Availability

Not applicable.
